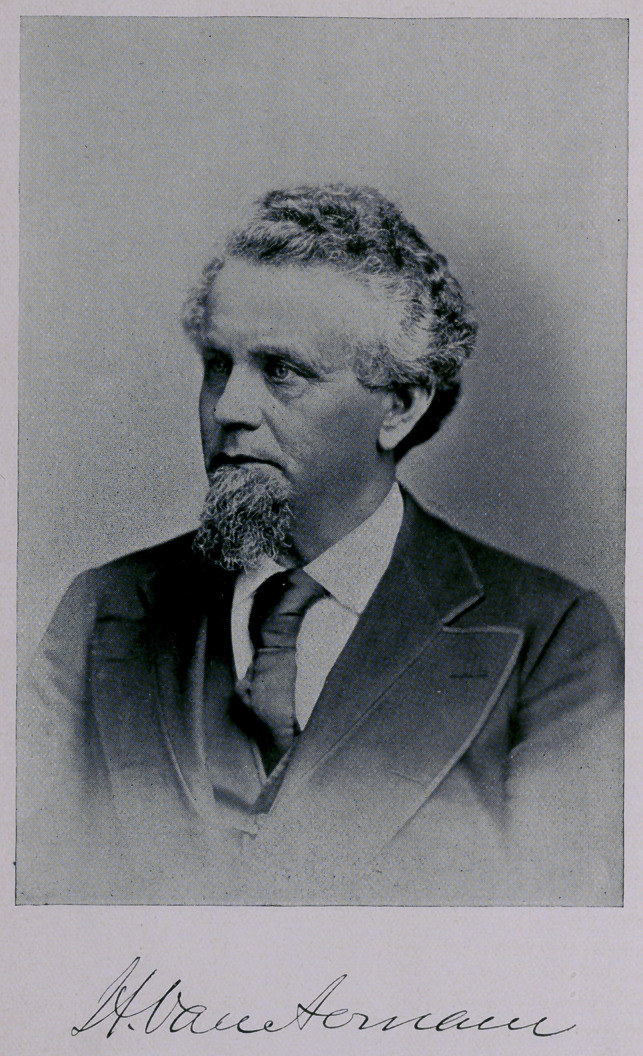# Hematoma of the Ovary1Read before the Gynecological and Obstetrical Society of Baltimore.

**Published:** 1894-07

**Authors:** George H. Rohé

**Affiliations:** Catonsville, Md.


### HEMATOMA OF THE OVARY.[1]

By GEORGE H. ROHÉ, M. D., Catonsville, Md.

Abdominal surgeons not infrequently find, in extirpated ovaries, small blood-clots, varying in size from a pea to a hazel nut. The nature of these clots seems not very clearly understood. In most cases they are believed to be due to excessive hemorrhage into the Graafian follicle after rupture, and the escape of the ovule. This view seems to me not tenable, because in not a few instances no rupture of the follicle has occurred. Besides, the corpus luteum, the successor of the ovule in the occupancy of the Graafian follicle, frequently contains no blood. Indeed, the view seems not irrational that hematoma of the ovary, no matter how small it may be, should always be regarded as a pathological formation, having no essential connection with the physiological process of ovulation. In such a specimen as that here shown, in which the blood-clot in the fresh state of the specimen was as large as a small chestnut, we have to deal with a pathological condition. The specimen is from a case of hystero-epilepsy of over eight years' duration, in which both ovaries and tubes were removed by abdominal section in 1891. The patient recovered, and has had no recurrence of the

1. Read before the Gynecological and Obstetrical Society of Baltimore.

epileptic attacks for over two years. Ovaries presenting this appearance are not rarely seen in abdominal section. I am informed that some surgeons simply extirpate the hematoma, stitch up the wound in the ovary, and drop the organ back into the pelvis. I may be permitted to express doubt whether any good purpose is served by this so-called "conservative" surgery. In all cases of this kind that have come under my notice, there were either adhesions or displacements of the ovaries, which are among the recognized indications for removal of these organs. Dr. B. F. Baer, who is known as a very careful and conservative surgeon, says in reference to these cases:[1] "Diseased ovaries, when due to hemorrhage into the Graafian follicles to such an extent as to produce the condition known as ovarian hematoma, should be removed. They cause intense suffering and there is no other means of relief."

Dr. Mary A. Dixon Jones, of Brooklyn, and Dr. Francis Foerster are of the opinion that hematoma of the ovary is preceded by conditions termed by them "Gyroma" and "Endothelioma." Indeed, the latter writer, basing his opinion upon somewhat extended microscopical study of ovaries, normal and pathological, claims that "what previously was called a corpus luteum is invariably an endothelioma." That the corpus luteum is an endothelial structure, may be accepted without dispute; that it should be called by a name heretofore applied to a malignant new formation, or that the consequences attributed by Foerster to this body hitherto considered so innocent, really follow in many cases, is, I think, open to grave doubt. Chronic oöphoritis and peri-oöphoritis, endarteritis and sclerosis are mentioned as histological findings, and pain and distress as clinical manifestations due to ovaries undergoing these morbid changes.

Dr. Foerster connects the corpora lutea with the production of hematoma as follows: "In my own experience a large number of so-called corpora lutea of menstruation are endotheliomata of a pathological type. They grow under the influence of a chronic oöphoritis without coming to a typical end, or gradually increasing in bulk and frequently leading to the formation of hematoma under incessant local and constitutional trouble.

It will, I think, be generally conceded that a hemorrhage into an ovarian follicle, or into ovarian stroma, does not take place when the ovary or the blood-vessels preserve their normal structural integrity. Some nutritional change must have preceded the hemorrhage. It is most reasonable to believe that this change is in the blood-vessels of the ovary. Whether this nutritional disturbance is due to new formations properly dignified by the names "gyroma" and "endothelioma," or whether it is simply the result of chronic inflammation, is a question that must be referred to the pathologists for further investigation. Rollin[2], who has recently made a study of ovarian hematoma, gives chronic oöphoritis as a local condition antedating the hemorrhage.

While the occurrence of small collections of blood in the Graafian follicles and minute extravasations in the ovarian stroma is not infrequent, the cases of so-called ovarian apoplexy, where the entire ovary is converted into a blood-cyst, varying from a billiard-ball to a fetal head in size, are much more rare. The case presently to be related shows, however, that there is no essential difference between the two classes of cases.

The case referred to is as follows:

E. L., born in United States, white, aged 21 years, single, was admitted to the Maryland Hospital for the Insane, November 18, 1893. Until a month before admission there had been no mental disturbance beyond a few hysterical attacks of varying severity, sometimes accompanied by convulsions. Her disposition was usually amiable, although she was of rather unstable temper. Her habits were always industrious. So far as was ascertained there was no hereditary pre-disposition to insanity. The hysterical outbreaks were usually coincident with the menstrual periods, and have only been present for the past four or five years. Up to a year ago her physical condition was very good, but for three years she has suffered with a good deal of pain during the catamenia. About a year ago she consulted a gynecologist, under whose care she remained for several months with apparent improvement. During the last three or four weeks before admission, a great change in her behavior was noticed. She became exalted, talkative, silly in conversation and action. When admitted, she carried a large doll, which she caressed and talked to in a childish manner. She was neat and cleanly in dress and habits and never noisy or maniacal. No apparent sexual excitement. At the end of two weeks she had lost all her delusions and was apparently restored to her normal mental condition. At the approach of the next menstrual period she became hysterical, had several convulsions, foamed at the mouth, screamed, or lay with eyes staring or closed. Reflexes normal. During these attacks she was unquestionably conscious of what was going on around her. One evening she set fire to her clothing, but the fire was promptly extinguished, and only a slight superficial reddening of small areas of the skin was produced. No serious results followed this attempt at self-destruction.

After the period was over, her normal mental condition returned, but she did not improve physically. She lost appetite, had nausea and became thin and anemic.

The pains in the iliac region persisted and became especially severe on the left side. Occipital headache, rhachialgia and pains in the limbs, with attacks of nausea and vomiting, were also present.

On January 18, 1894, a vaginal examination demonstrated an elastic swelling behind and to the left of the uterus, which was exquisitely sensitive to the touch. To the right there appeared to be an enlarged and prolapsed ovary. The uterus was adherent posteriorly, but somewhat movable.

The clinical diagnosis of adherent uterus, prolapsed ovary on the right and cystic ovary or ovarian abscess on the left side was made, and an operation for the relief of these conditions recommended to her, and her consent readily obtained. Inasmuch as she was, and had been for some weeks, entirely rational, her own consent was considered sufficient authority to proceed.

Abdominal section was done on January 28, 1894. Passing two fingers through the incision down to the fundus, this was found adherent, the tubes and ovaries on both sides being also bound down by adhesions. After carefully separating the latter, the right ovary, enlarged to the size of an English walnut, was brought up, ligated together with the thickened tube close to the uterus, and removed. In place of the left ovary was a cystic tumor as large as a mandarin orange, which ruptured as it was brought out of the abdominal wound, and discharged a lot of softly-coagulated blood. My first thought was of an ectopic pregnancy, but, as an examination of the specimen will show, this was a mistake and an unjust suspicion. After the tube and remains of the cyst were ligated and removed, the peritoneal cavity was flushed out with hot, distilled water, and the abdominal wound closed with silkworm gut sutures. No drainage.

The subsequent course was uneventful, except that on the second day the temperature rose to 101 degrees F., and the pulse to 102. After a purgative enema of magnesium sulphate and glycerine, this slight disturbance vanished.

The stitches were removed on the seventh day and the wound was found dry and thoroughly united. Patient out of bed on the twenty-first day.

Since the operation the patient has suffered no pain, is cheerful and industrious, not hysterical and has gained flesh. Her mental condition apparently normal. The patient was discharged entirely recovered March 15, 1894.

The walls of the blood cyst are apparently composed of ovarian stroma; the tube is somewhat thickened, but contains no pus.

The right ovary, on section, shows two blood-clots about the size of hazel nuts, apparently occupying unruptured Graafian follicles. This case seems to show, on the two sides, examples of two forms of ovarian hematoma which are, however, rarely associated in the same individual. If any conclusion can be drawn from a single case, it is that the rather common follicular hematoma and the infrequent ovarian apoplexy are identical in origin.

Winckel[3] refers to three cases of follicular hemorrhage into the ovaries after severe burns. The burn which my patient received about a month before the operation might be considered suggestive, if it had been more serious. The firm adhesions were, however, evidence of a longer duration, at least of the local inflammatory condition.

Of the more recent cases reported, is one by Doran in Vol. XXXII. of the Transactions of the London Obstetrical Society. Doran considered it a hemorrhage into the ovarian stroma from rupture of a follicle. The cyst wall was one-eighth of an inch thick and consisted of ovarian stroma. Dr. Mundé[4] briefly reports a case of hematoma of both ovaries, one being the size of an orange and the other of a hen's egg. Dr. E. E. Montgomery,[5] in commenting on this case, refers to a similar one under his observation. Duncan reports a case in which there was hematosalpinx in connection with the ovarian hematoma. The history of the case suggests ectopic pregnancy, which seems, however, to have been excluded.

I am reminded here of a case which I saw about twelve years ago in the service of late Dr. A. F. Erich at the Maryland Woman's Hospital. The patient was a white, single woman, 35 years of age. The tumor, supposed to be an ovarian cystoma, was about the size of a fetal head, and when brought to the abdominal incision and tapped with the trocar, thick, black blood was evacuated. The patient died of purulent peritonitis about the fifth day, and at the autopsy a perforation of the rectum was found. How this was produced could not be cleared up. It may have been torn through in separating adhesions. A number of apparently similar cases, in which the cyst ruptured and caused death from septic peritonitis, are recorded by Bernutz and Goupil, but most of these were probably cases of extra-uterine pregnancy.

An ovarian hematoma may rupture and give rise to a pelvic hematocele. In other cases the bleeding may continue and the patient die of hemorrhage. The most serious danger from rupture is, however, peritonitis and sepsis. I am informed by Dr. Joseph

Price that the contents of an ovarian hematoma are usually exceedingly virulent and liable to cause septic peritonitis, if the blood-cyst is allowed to rupture within the peritoneal cavity.

The diagnosis of ovarian hematoma cannot be definitely made before abdominal section. Even when rupture occurs and a hematocele is formed, the diagnosis rests between several conditions, often differentiated with the greatest difficulty, even after operation.

The only rationally indicated procedure is removal of the affected organ by abdominal section.

REFERENCES.

1. Proceedings Philadelphia Obstetrical Society, June 12, 1892.
2. Frauen Krankheiten, 2 Aufl., p. 700.
3. *American Journal of Obstetrics*, June, 1890, p. 638.
4. Sajous' Annual, 1891, II., G. 46.
5. Ibid, 1893, II., G. 5.

—*Maryland Medical Journal.*

## Figures and Tables

**Figure f1:**